# Incidence and risk factors of venous thromboembolism following radical nephrectomy: A meta-analysis of observational studies

**DOI:** 10.1080/20905998.2025.2457923

**Published:** 2025-01-26

**Authors:** Yuan Li, Chiming Gu, Siyi Li, Franky Leung Chan, Shusheng Wang

**Affiliations:** a Department of Urology, The Second Affiliated Hospital of Guangzhou University of Chinese Medicine, Guangzhou, China; b School of Biomedical Sciences, The Chinese University of Hong Kong, Shatin, Hong Kong, China

**Keywords:** Radical nephrectomy, risk factor, venous thromboembolism, incidence, meta-analysis

## Abstract

**Background:**

A meta-analysis was conducted to evaluate the incidence of venous thromboembolism (VTE) following radical nephrectomy and to examine the related risk factors.

**Methods:**

We conducted a comprehensive search for primary research articles from the inception of the databases up to October, 2024 across several databases, including MEDLINE, Embase, and the Cochrane Library. We employed random effects models to determine multivariate adjusted odds ratios (ORs) along with their corresponding 95% confidence intervals (CIs).

**Results:**

In total, 11 studies including 140,795 patients who underwent radical nephrectomy met inclusion criteria. The results showed that postoperative VTE incidence was 1.01% (95% CI = 1.01–1.02; *p* < 0.001) within 30 days. Moreover, diabetes, open nephrectomy, and history of VTE were related to higher odds of VTE through regression analysis. Analyses of sensitivity and meta-regression demonstrated the robustness of the study’s findings.

**Conclusions:**

The overall incidence of VTE after radical nephrectomy is 1%, occurring approximately one month after surgery, and may be related to factors such as open surgery, diabetes and history of VTE. This reminds urologists that more aggressive thromboprophylaxis may be required for these patients.

**PROSPERO Registration number:**

CRD42023439919.

## Introduction

Venous thromboembolism (VTE) after surgery, comprising pulmonary embolism (PE) and deep vein thrombosis (DVT), is characterized by high treatment costs, long duration, and can lead to irreversible health, quality of life decline, and even death. It is a major clinical and socio-economic burden. Annually, an estimated 60,000 to 100,000 Americans succumb to VTE, with approximately 10–30% passing away within a month after a prostate cancer diagnosis [1]. In particular, about 25% of people with PE may die suddenly [2]. Several studies have shown that postoperative VTE is related to lower morbidity and mortality, although the incidence of postoperative VTE varies by cancer subtype [3–6]. Among the many urological procedures that have been conducted, prostatectomy (1.0%) and cystectomy (2.9%) have high rates of postoperative VTE [7]. Studies have been performed to clarify the risk factors related to VTE after cystectomy and prostatectomy [8,9].

However, data on risk factors related to VTE after nephrectomy are scarce. Hence, we sought to identify potential risk factors related to VTE after radical nephrectomy and the incidence of VTE events after these procedures, potentially having important clinical implications for prevention strategies and clinical practice measures.

## Materials and methods

In line with the PRISMA guidelines [10], this meta-analysis of observational studies was conducted. Consequently, there was no necessity for ethical approval or informed consent from patients.

### Literature search

We sourced relevant studies from several databases, including Embase, MEDLINE, and the Cochrane Library, from the inception of the databases up to October, 2024. These studies investigated the correlation between the occurrence and risk factors of postoperative VTE following radical nephrectomy for kidney cancer. The database search encompassed all languages, publication types, and regions. The search strategy included a mix of Medical Subject Headings (MeSH) and non-MeSH terms, detailed as follows: (‘nephrectomy’ OR ‘nephrectomies’) AND (‘incidence’) AND (‘risk factor’). We also manually reviewed the bibliographies of past reviews and related articles to locate additional pertinent reports. Any discrepancies were resolved through agreement with the co-investigators.

### Inclusion and exclusion criteria

First, two reviewers separately examined the titles and summaries of the initial studies to eliminate any that failed to meet the specified criteria, documenting the reasons for their exclusion. Subsequently, a detailed review of the full texts of potentially eligible studies was performed. Studies that fulfilled the following eligibility criteria were included: (1) original researches regarding the correlation between the incidence of postoperative VTE (DVT or PE) and/or associated risk factors after radical nephrectomy (i.e. open nephrectomy or laparoscopic nephrectomy with or without lymph node dissection) in patients with kidney cancer; (2) studies that included risk estimates (such as OR, hazard ratio [HR], and relative risk) with their 95% confidence intervals (CIs); if these were not provided, adequate raw data for calculation was necessary; and (3) observational research (including case-control, prospective or retrospective cohort, or cross-sectional studies) published as original articles. When duplicate publications were encountered, only the latest or highest-quality study was considered. Case reports, letters, reviews, and conference abstracts were excluded. Any conflicts were resolved through consensus among the co-investigators.

### Data extraction and methodological quality evaluation

Two researchers independently gathered crucial data from the selected studies. The extracted details were organized into a standardized Excel file, including the first author, publication year, data source, country, sample size, study design, surgical procedure, participant characteristics (such as follow-up duration and average age), VTE incidence and/or risk factors, risk estimates with 95% CIs, and confounders. If any studies lacked sufficient information, we reached out to the lead author to obtain the missing data. Any disagreements during data extraction were resolved through consensus with the co-investigators.

The quality of the included studies was independently evaluated by two authors using the Newcastle – Ottawa Scale (NOS) [11], which includes 10 criteria. Each criterion was rated as ‘yes’ (scoring 1) or ‘no/unclear’ (scoring 0) based on the extracted study information. The overall score, ranging from 0 to 10, classified the studies as follows: scores of 8–10 indicated high quality, 5–7 indicated moderate quality, and scores below 5 indicated low quality. Any disagreements were resolved through discussion among the co-researchers.

### Data integration and analysis

**Figure 1.**
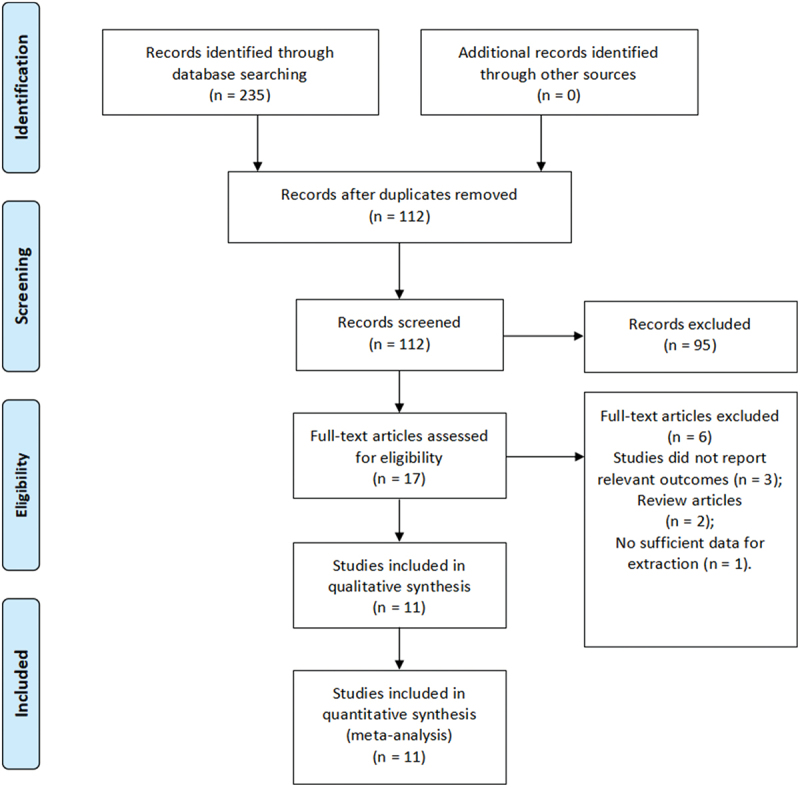
Flow diagram of literature searches according to the preferred reporting items for systematic reviews and meta-analyses statement.

Total risk estimates for the study were calculated using ORs and associated 95% CIs using STATA statistical software (version 15.0; serial number: 10699393; StataCorp Wyb) to appraise the association between the incidence and related risk factors of postoperative VTE after radical nephrectomy for kidney cancer. The flowchart (Figure 1) was generated from the PRISMA Flow Diagram table to show the process of searching and screening literature more easily [10]. Forest plots illustrate the aggregate findings and variability across studies. To assess the influence of variability on the results of the meta-analysis, an I [2] test was conducted. Various I-values indicate varying levels of heterogeneity. According to Cochrane review guidelines [12], a random effects model was applied when heterogeneity was I [2] ≥ 50%; otherwise, a fixed-effects model was used. A threshold of *p* < 0.05 was established to determine statistical significance. Subgroup analyses by country and study design were performed to investigate how different methodologies and patient characteristics influenced heterogeneity. Additionally, a sensitivity analysis was conducted by excluding each study one at a time to test the robustness and consistency of the results. Meta-regression analyses, using the restricted maximum likelihood method, were carried out to identify potential sources of heterogeneity across various variables. Finally, Egger’s test was employed to assess publication bias [13].

## Results

### Study identification and selection

Our initial search yielded 235 records. After removing duplicates, 112 studies remained. Following a title and abstract screening, 95 studies were excluded, leaving 17 articles for full-text evaluation. Ultimately, 6 articles were excluded for the following reasons: three studies lacked relevant outcomes, and two were review articles; one study did not have enough data to extract (as shown in Figure 1). Eventually, 11 observational studies [7,14–23] comprising 140,795 patients who underwent radical nephrectomy were included for this meta-analysis in accordance with the inclusion criteria.

### Study characteristics

Table 1 outlines the fundamental characteristics of the included studies. All of these studies are observational in nature, with ten being retrospective [7,14,15,17–23] and one being prospective [16], published between 2006 and 2020. The sample sizes varied from 170 to 108,430 patients. The reported incidence of VTE following radical nephrectomy ranged from 0.7% to 1.7%. Six of the studies were conducted in the United States; two were conducted in Canada [17,23]; three studies were conducted in Denmark [15], France [16], and France [20], respectively. Only two studies [15,18] explicitly identified the risk factors for VTE following radical nephrectomy. All the selected studies were published in English. Importantly, each included study provided risk estimates that were adjusted for confounding variables.

**Table 1.** Characteristics of the included studies.

First author year	Study design	Country	Database source (Duration)	Sample size	Mean age, years	Incidence, %	Follow-up period, days
Alberts BD. 2014	RS	America	ACS-NSQIP database (2005–2012)	4568	NA	1.2	30 days
Azawi NH. 2020	RS	Denmark	Danish Renal Cancer Database (2010–2018)	5213	64 (54–71)	1	30 days
Clément C. 2011	PS	France	Department of Urology at North Hospital, Marseille, France (2005–2009)	177	62.5 ± 9	0.7	30 days
Ihaddadene R. 2014	RS	Canada	Ottawa Hospital (2005–2012)	170	60 (22–88)	1.2	2 years
Jordan BJ. 2017	RS	America	ACS-NSQIP database (2006–2012)	7198	62.2 ± 13.8	1.3	30 days
Mukherjee D. 2008	RS	America	Nationwide Inpatient Sample (2001–2005)	7191	59	0.85	30 days
Park H. 2019	RS	South Korea	Seoul National University Hospital (1999–2005)	2762	56 (10–87)	1	39 months
Patel HV. 2022	RS	America	Premier Healthcare database (2013–2017)	108,430	62	1.2	90 days
Pettus JA. 2006	RS	America	Renal tumor database (1989–2005)	2208	62.6 (53.2–70.5)	1.5	30 days
Tyson MD. 2014	RS	America	NSQIP database (2005–2011)	2702	62 (51–71)	1.67	30 days
Yokom DW. 2014	RS	Canada	Ottawa Hospital (2005–2012)	176	62.5	1.7	30 days

ACS-NSQIP, American College of Surgeons National Surgical Quality Improvement Program; NSQIP, National Surgical Quality Improvement Program; NA, not available; RS, Retrospective study; PS, Prospective study.

### Methodological quality assessment

The methodological quality of the included studies was assessed using the NOS. Two studies [15,18] scored nine or ten points, indicating high quality. Eight studies [7,16,17,19–23] received seven or eight points, classifying them as moderate quality. The remaining study [14] scored five points and was deemed to be of low quality.

### The incidence and risk factors of VTE after radical nephrectomy

**Figure 2.**
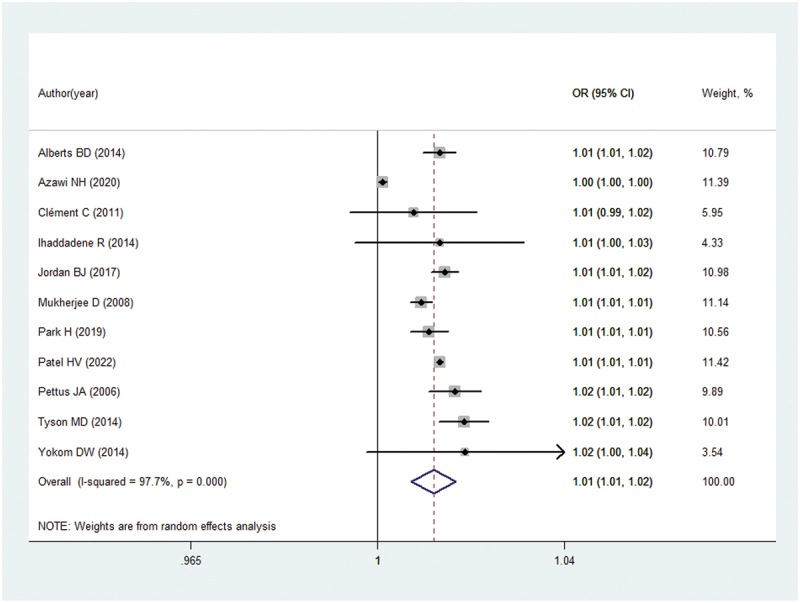
Meta-analysis on the incidence of VTE after radical nephrectomy. CI, confidence interval, OR, odds ratio.

**Figure 3.**
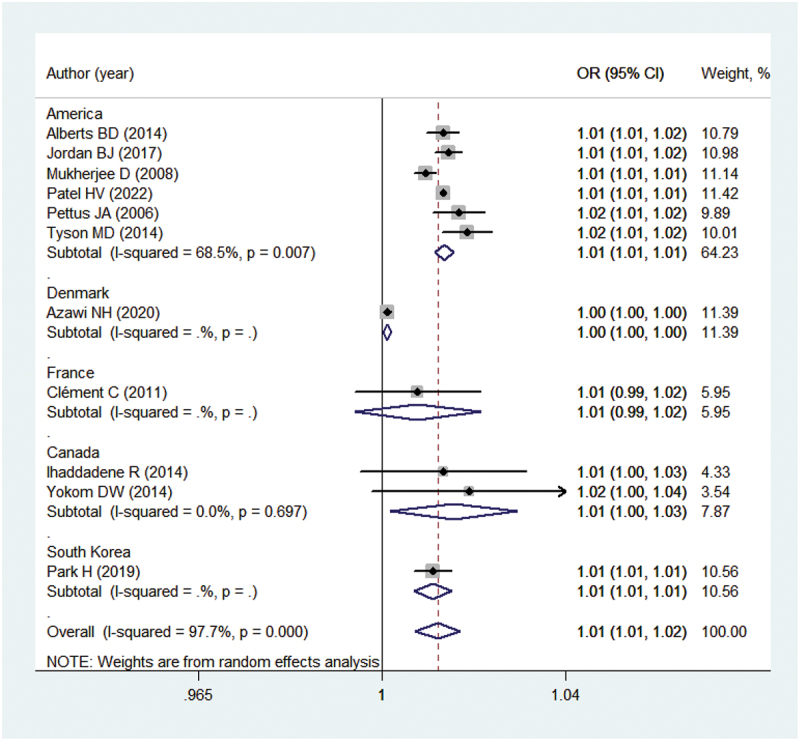
Results of subgroup analyses. CI, confidence interval, OR, odds ratio.

Eleven studies [7,14–23] offered adequate data on the relationship between the incidence and risk factors of postoperative VTE following radical nephrectomy for kidney cancer. The findings indicated that the overall postoperative VTE rate was 1.01% (95% CI = 1.01–1.02; *p* < 0.001) within 30 days after the procedure, after adjusting for confounding variables. Due to significant heterogeneity, a random effects model was employed for the pooled analysis, as depicted in Figure 2. Moreover, our subgroup analyses revealed that the pooled incidence rates are all statistically significant when stratifying studies by different countries except for the studies performed in France (as shown in Figure 3). Importantly, the benefit of the subgroup analysis lies in the potential to identify variations in outcomes that may be influenced by regional factors such as environmental conditions, healthcare access, cultural differences, and population demographics. Although the risk factors were reported in only two studies [15,18] with limited data which could not be pooled, it still needs to be stated that diabetes (OR = 1.61, 95% CI = 1.05–2.48; *p* < 0.05), open nephrectomy (OR = 3.33, 95% CI = 1.69–6.55; *p* < 0.05), and history of VTE (OR = 26.3, 95% CI = 14.2–48.9; *p* < 0.05) were related to the higher odds of VTE through regression analysis.

**Figure 4.**
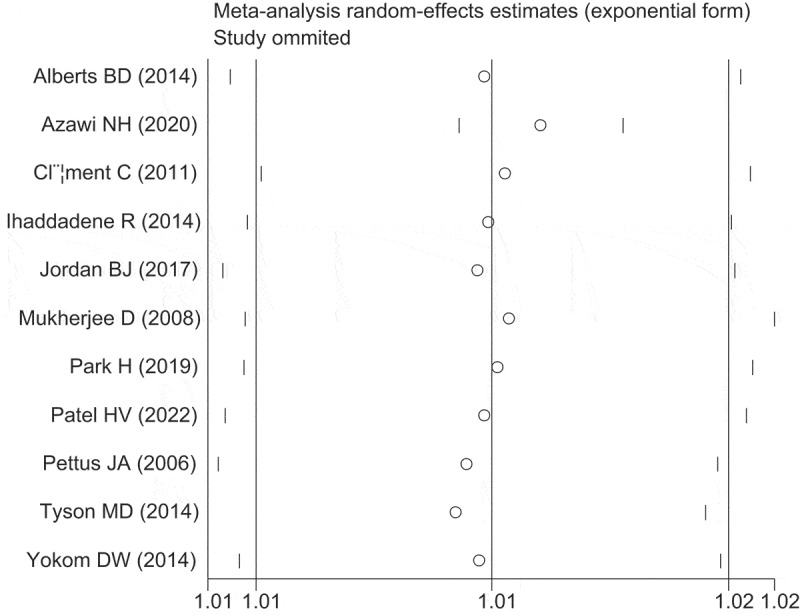
Results of sensitivity analyses.

**Figure 5.**
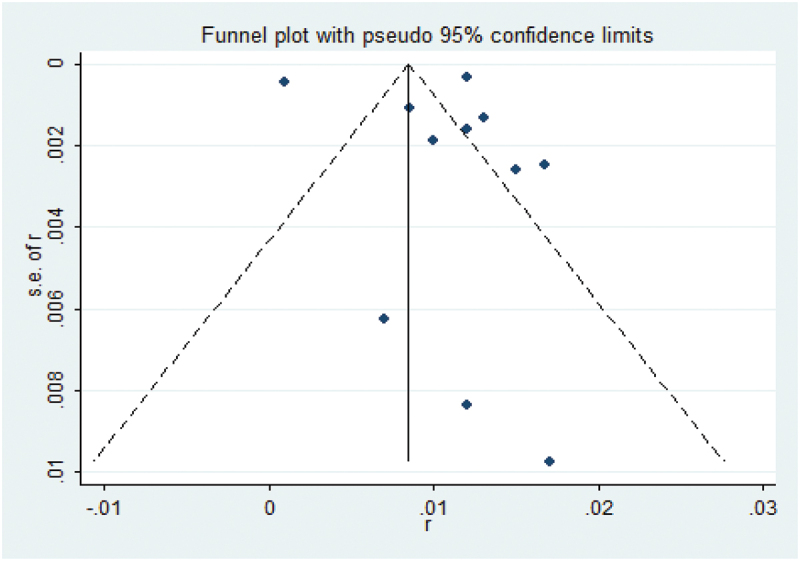
Results of Egger’s test with funnel plot.

Sensitivity analyses revealed that excluding each study individually did not significantly alter the overall results (Figure 4). To further investigate the observed heterogeneity among studies, a meta-regression analysis was conducted. The analysis indicated that none of the covariates, including country (*p* = 0.962), contributed to the heterogeneity. The adjusted R-squared value indicates the proportion of variance in the response variable that can be explained by the regression model, after accounting for the number of predictors used. In this case, a value of 0.62% suggests that the regression variables have a minimal effect on explaining the variability of the response variables. Lastly, Egger’s test results showed no evidence of publication bias, as indicated by the formal statistical test (Egger’s test, *p* = 0.690) (Figure 5).

## Discussion

### Main findings

This meta-analysis investigated the link between the occurrence and associated risk factors of postoperative VTE in patients undergoing radical nephrectomy for kidney cancer. The results indicated that the overall incidence of VTE after radical nephrectomy is low, occurring approximately one month after surgery, and may be related to factors such as open surgery, diabetes and history of VTE. Interestingly, sensitivity analyses indicated that the overall stability of the findings remained consistent despite the exclusion of individual studies. Although the meta-regression failed to pinpoint specific factors contributing to inter-study heterogeneity, no evidence of publication bias was detected based on Egger’s test and the funnel plot analysis.

We observed that two studies explored the risk factors associated with VTE amongst the included studies [15,18]. Azawi et al. [15] conducted a retrospective study that comprise 5,213 patients who underwent radical nephrectomy. They found that postoperative VTE is rare (1%). The likelihood of postoperative VTE is heightened by factors such as a history of VTE (OR = 13.3, *p* < 0.001) and open nephrectomy (OR = 2.5, *p* = 0.001). Moreover, lymph node dissection (OR = 2.0, *p* = 0.04) and the duration of hospital stay (OR = 0.98, *p* = 0.02) share a similar relationship. These factors should be taken into account when devising a comprehensive VTE treatment plan. In addition, Jordan *et al* [18]. found that for patients undergoing radical nephrectomy, the combined rate of VTE was 1.3%. Several factors have been associated with a higher incidence of VTE, including longer operative times, open surgical approaches, and patients with preoperative respiratory distress. We expect more studies focusing on this issue to be published in the future to guide clinical practice.

### Implications for clinical practice

This study presents a comprehensive meta-analysis highlighting the necessity for urologists to prioritize VTE prevention following radical nephrectomy. Our findings reveal that the incidence of VTE events within one month post-surgery is relatively low, at 1%. According to EAU guidelines, standardized VTE management should include daily administration of tinzaparin anti-Xa 4500 IE or dalteparin anti-Xa 5000 IE, starting from the first preoperative day until discharge [24]. Tikkinen et al. [25] analyzed the risk of VTE over time in patients undergoing urological surgery, discovering that the risk increased up to four weeks post-surgery before stabilizing. Furthermore, in a systematic review and meta-analysis aligned with EAU guidelines on VTE prevention, Tikkinen et al. [26] assessed the one-month VTE risk following non-oncologic renal surgery, categorizing patients into various risk groups. However, only 40% of the included studies reported details of VTE prophylaxis strategies, with a combined estimate of 2.9 days (IQR 1.9–4.0) for the use of VTE prophylaxis. Our study examined several risk factors, including diabetes mellitus, open surgery, and a history of thrombosis. We anticipate future research that will validate our findings and investigate additional risk factors, ultimately contributing to the development of improved treatment strategies for these patients. By identifying a risk factor for VTE in patients undergoing radical nephrectomy, we suggest that personalized VTE prevention strategies could be effectively implemented. Further research is needed to enhance our understanding of how to customize both short-term and long-term VTE prevention strategies for high-risk patients. For patients with one or more risk factors, preoperative/or postoperative thromboprophylaxis may be required. the ACS-NSQIP has generated a surgical risk calculator that can be accessed online and our definitions are in line with the currently available risk calculators [27]. Due to the high incidence of postoperative VTE, patients undergoing radical cystectomy are typically prescribed a continuous 4-week course of pharmacological thromboprophylaxis [28]. A recent study indicated that administering a single preoperative dose of thromboprophylaxis in patients undergoing major cancer surgeries, such as radical nephrectomy, did not increase bleeding or transfusion rates but significantly reduced the incidence of VTE [29]. With these factors in mind, a four-week course of thromboprophylaxis after radical nephrectomy is expected to be evaluated in future in patients with high risk of thrombosis and whether it should be used aggressively to reduce mortality due to postoperative VTE.

### Strength and limitations

This meta-analysis offers several key advantages. Firstly, it is the first to investigate the correlation between the incidence and risk factors of postoperative VTE following radical nephrectomy in kidney cancer patients, with subgroup analyses conducted by different countries to assess the impact of these variables on heterogeneity, following PRISMA guidelines. Secondly, to minimize the influence of confounding factors on the overall results, multivariate-adjusted risk estimates were employed. Lastly, the robustness and validity of our findings were confirmed through meta-regression and sensitivity analyses.

However, it is important to recognize several limitations of this meta-analysis. Firstly, nearly all included studies are retrospective, which may introduce disadvantages such as missing data and potential bias. Secondly, incomplete retrieval due to non-extractable data and unavailable full texts is another concern. Thirdly, significant heterogeneity was observed, and despite conducting a meta-regression, the risk of introducing substantial heterogeneity remains. Lastly, our understanding of the risk factors associated with VTE after radical nephrectomy is limited, as these crucial details were inadequately reported in the included studies. Therefore, further high-quality research is necessary to enhance the predictive power of potential risk factors.

## Conclusion

The current evidence suggests that the overall incidence of VTE after radical nephrectomy is 1%, occurring approximately one month after surgery. Moreover, open surgery, diabetes and history of VTE may increased the probability of postoperative VTE. Taken together, these findings have important implications for further research into the role of preoperative screening of patients with VTE, preoperative counselling of patients with VTE and risk stratification for thromboprophylaxis before and after radical nephrectomy.

## Data Availability

The datasets used in this study are available from the corresponding authors on reasonable request.

## Abbreviations

VTE: venous thromboembolism
ORs: odds ratios
DVT: deep vein thrombosis
MeSH: Medical Subject Headings
HR: hazard ratio
NOS: Newcastle – Ottawa Scale

## References

[1] Heit JA, Silverstein MD, Mohr DN, et al. Predictors of survival after deep vein thrombosis and pulmonary embolism: a population-based, cohort study. Arch Intern Med. 1999;159(5):445–453. doi: 10.1001/archinte.159.5.445 10074952

[2] Kakkar AK, Haas S, Wolf H, et al. Evaluation of perioperative fatal pulmonary embolism and death in cancer surgical patients: the MC-4 cancer substudy. Thromb Haemost. 2005;94(10):867–871. doi: 10.1160/TH04-03-0189 16270644

[3] Heath OM, van Beekhuizen HJ, Nama V, et al. Venous thromboembolism at time of diagnosis of ovarian cancer: survival differs in symptomatic and asymptomatic cases. Thromb Res. 2016;137:30–35. doi: 10.1016/j.thromres.2015.11.030 26653367

[4] Kumar A, Hurtt CC, Cliby WA, et al. Concomitant venous thromboembolism at the time of primary EOC diagnosis: perioperative outcomes and survival analyses. Gynecol Oncol. 2017;147(3):514–520. doi: 10.1016/j.ygyno.2017.09.020 28947173

[5] Gainsbury ML, Erdrich J, Taubman D, et al. Prevalence and predictors of preoperative venous thromboembolism in asymptomatic patients undergoing major oncologic surgery. Ann Surg Oncol. 2018;25(6):1640–1645. doi: 10.1245/s10434-018-6461-2 29626305

[6] Schomburg JL, Krishna S, Cotter KJ, et al. Preoperative incidence of deep venous thrombosis in patients with bladder cancer undergoing radical cystectomy. Urology. 2018;116:120–124. doi: 10.1016/j.urology.2018.01.052 29551621

[7] Tyson MD, Castle EP, Humphreys MR, et al. Venous thromboembolism after urological surgery. J Urol. 2014;192(3):793–797. doi: 10.1016/j.juro.2014.02.092 24594402

[8] Sun AJ, Djaladat H, Schuckman A, et al. Venous thromboembolism following radical cystectomy: significant predictors, comparison of different anticoagulants and timing of events. J Urol. 2015;193(2):565–569. doi: 10.1016/j.juro.2014.08.085 25150642

[9] Tyritzis SI, Wallerstedt A, Steineck G, et al. LAPPRO steering committee. Thromboembolic complications in 3,544 patients undergoing radical prostatectomy with or without lymph node dissection. J Urol. 2015;193(1):117–125. doi: 10.1016/j.juro.2014.08.091 25158271

[10] Moher D, Shamseer L, Clarke M, et al. Preferred reporting items for systematic review and meta-analysis protocols (PRISMA-P) 2015 statement. Syst Rev. 2015;4(1):1. doi: 10.1186/2046-4053-4-1 25554246 PMC4320440

[11] Wells GA, Shea B, O’Connell D, et al. The Newcastle-Ottawa Scale (NOS) for assessing the quality of nonrandomized studies in meta-analysis. Ottawa hospital research institute website. 2014. Available from: http://www.ohri.ca/programs/clinical_epidemiology/oxford.asp

[12] Higgins JPT, Green S. Cochrane handbook for systematic reviews of interventions version 5.1.0 (updated March 2011). Cochrane Collab. 2011.

[13] Egger M, Davey Smith G, Schneider M, et al. Bias in meta-analysis detected by a simple, graphical test. BMJ. 1997;315(7109):629–634. doi: 10.1136/bmj.315.7109.629 9310563 PMC2127453

[14] Alberts BD, Woldu SL, Weinberg AC, et al. Venous thromboembolism after major urologic oncology surgery: a focus on the incidence and timing of thromboembolic events after 27,455 operations. Urology. 2014;84(4):799–807. doi: 10.1016/j.urology.2014.05.055 25156513

[15] Azawi NH, Subhi Y, Tolouee S, et al. Incidence and associated risk factors of venous thromboembolism after open and laparoscopic nephrectomy in patients administered short-period thromboprophylaxis: a Danish nationwide population-based cohort study. Urology. 2020;143:112–116. doi: 10.1016/j.urology.2020.06.007 32569656

[16] Clément C, Rossi P, Aissi K, et al. Incidence, risk profile and morphological pattern of lower extremity venous thromboembolism after urological cancer surgery. J Urol. 2011;186(6):2293–2297. doi: 10.1016/j.juro.2011.07.074 22014814

[17] Ihaddadene R, Yokom DW, Le Gal G, et al. The risk of venous thromboembolism in renal cell carcinoma patients with residual tumor thrombus. J Thromb Haemost. 2014;12(6):855–859. doi: 10.1111/jth.12580 24702743

[18] Jordan BJ, Matulewicz RS, Trihn B, et al. Venous thromboembolism after nephrectomy: incidence, timing and associated risk factors from a national multi-institutional database. World J Urol. 2017;35(11):1713–1719. doi: 10.1007/s00345-017-2046-0 28516316

[19] Mukherjee D, Lidor AO, Chu KM, et al. Postoperative venous thromboembolism rates vary significantly after different types of major abdominal operations. J Gastrointest Surg. 2008;12(11):2015–2022. doi: 10.1007/s11605-008-0600-1 18668299

[20] Park H, Jeong CW, Yuk H, et al. Influence of tumor thrombus on occurrence of distant venous thromboembolism and survival in patients with renal cell carcinoma after surgery. Clin Appl Thromb Hemost. 2019;25:1076029618823288. doi: 10.1177/1076029618823288 30808214 PMC6714931

[21] Patel HV, Sterling JA, Srivastava A, et al. The impact of venous thromboembolism on mortality and morbidity during nephrectomy for renal mass. Urology. 2022;168:122–128. doi: 10.1016/j.urology.2022.05.033 35691439

[22] Pettus JA, Eggener SE, Shabsigh A, et al. Perioperative clinical thromboembolic events after radical or partial nephrectomy. Urology. 2006;68(5):988–992. doi: 10.1016/j.urology.2006.06.026 17113889

[23] Yokom DW, Ihaddadene R, Moretto P, et al. Increased risk of preoperative venous thromboembolism in patients with renal cell carcinoma and tumor thrombus. J Thromb Haemost. 2014;12(2):169–171. doi: 10.1111/jth.12459 24283651 PMC4238732

[24] Ljungberg B, Albiges L, Abu-Ghanem Y, et al. European Association of Urology guidelines on Renal Cell Carcinoma: the 2022 update. Eur Urol. 2022;82(4):399–410. doi: 10.1016/j.eururo.2022.03.006 35346519

[25] Tikkinen KAO, Agarwal A, Craigie S, et al. Systematic reviews of observational studies of risk of thrombosis and bleeding in urological surgery (ROTBUS): introduction and methodology. Syst Rev. 2014;3(1):150. doi: 10.1186/2046-4053-3-150 25540016 PMC4307154

[26] Tikkinen KAO, Craigie S, Agarwal A, et al. Procedure-specific risks of thrombosis and bleeding in urological non-cancer surgery: systematic review and meta-analysis. Eur Urol. 2018;73(2):236–241. doi: 10.1016/j.eururo.2017.02.025 28284738

[27] ACS NSQIP Surgical Risk Calculator. [c2007–2017 3/20/2017]. www.riskcalculator.facs.org

[28] Rasmussen MS. Preventing thromboembolic complications in cancer patients after surgery: a role for prolonged thromboprophylaxis. Cancer Treat Rev. 2002;28(3):141–144. doi: 10.1016/S0305-7372(02)00043-9 12234565

[29] Selby LV, Sovel M, Sjoberg DD, et al. MSKCC VTE task force. Preoperative chemoprophylaxis is safe in major oncology operations and effective at preventing venous thromboembolism. J Am Coll Surg. 2016;222(2):129–137. doi: 10.1016/j.jamcollsurg.2015.11.011 26711793 PMC4729628

